# Proline Enhances Resistance and Recovery of Oilseed Rape after a Simulated Prolonged Drought

**DOI:** 10.3390/plants12142718

**Published:** 2023-07-21

**Authors:** Sigita Jurkonienė, Rima Mockevičiūtė, Virgilija Gavelienė, Vaidevutis Šveikauskas, Mariam Zareyan, Elžbieta Jankovska-Bortkevič, Jurga Jankauskienė, Tautvydas Žalnierius, Liudmyla Kozeko

**Affiliations:** 1 Laboratory of Plant Physiology, Nature Research Centre, Akademijos Str. 2, 08412 Vilnius, Lithuania; rima.mockeviciute@gamtc.lt (R.M.); virgilija.gaveliene@gamtc.lt (V.G.); vaidevutis.sveikauskas@gamtc.lt (V.Š.); elzbieta.jankovska@gamtc.lt (E.J.-B.); jurga.jankauskiene@gamtc.lt (J.J.); tautvydas.zalnierius@gamtc.lt (T.Ž.); 2 Department of Cell Biology and Anatomy, M.G. Kholodny Institute of Botany of the National Academy of Sciences of Ukraine, Tereshchenkivska Str. 2, 01601 Kyiv, Ukraine; liudmyla.kozeko@gmail.com

**Keywords:** *Brassica napus*, exogenous proline, recovered growth, simulated drought

## Abstract

This study was carried out to evaluate the effect of exogenous proline on the growth, biochemical responses, and plant recovery of drought-stressed oilseed rape plants after renewed irrigation. The experiment was conducted under controlled laboratory conditions. After 21 days of cultivation, 3–4 leaf stage seedlings were sprayed with proline (1 mM), then subjected to prolonged drought stress for 8 days to achieve a severe water deficit, next, irrigation was resumed and recovery was assessed after 4 days. The results show that exogenous application of proline reduced the drought-induced growth inhibition of seedlings while maintaining relative water content (RWC) and growth parameters closer to those of irrigated plants. Proline had a positive effect on chlorophyll accumulation and membrane permeability while decreasing ethylene, H_2_O_2_, and MDA levels. Moreover, after 4 days of recovery, the H_2_O_2_ content of the proline-treated plants was significantly lower (2-fold) and the MDA content was close to that of continuously irrigated plants. Thus, all these biochemical reactions influenced plant survival: after drought + proline treatment, the number of surviving plants was two times higher than that of drought-treated plants. The findings show that exogenous proline has antioxidant, osmotic, and growth-promoting properties that improve the drought tolerance of winter oilseed rape plants and is, therefore, beneficial for drought adaptation in oilseed rape.

## 1. Introduction

Drought stress has detrimental effects on plant growth and development and poses a major threat worldwide to sustainable crop production in a rapidly changing environment. Europe’s climate has already become more extreme than previously predicted and rainfall deficits can occur at any time of year [1,2,3]. Plants employ different physiological and molecular defenses to become tolerant to drought stress. It has been estimated that about 80–95% of the fresh biomass of the plant body is water, which plays a vital role in various physiological processes, including many aspects of plant development, and metabolism [4]. One of the adaptive metabolic responses to drought is the accumulation of proline [5]. Proline accumulation is a common physiological response in many plants to a variety of biotic and abiotic stresses [6,7]. Proline accumulates in the cytosol without damaging cellular structures, and it is an essential part of the physiological adaptations to stress in many plant species [8,9]. Therefore, proline may help plants to regulate the osmotic potential of cells and to improve water absorbance and translocation under drought conditions. Farooq et al. [10] reported that the use of proline as an osmotic protection against water deficit in wheat plants resulted in the accumulation of high levels of chlorophyll, proline, glycine betaine, and all soluble phenols. In addition, the use of osmoprotectants has attracted a lot of attention due to their high efficiency, ease of use, low cost, and lack of need for advanced equipment. Osmoprotectants or compatible solutions are small, highly soluble organic molecules at physiological pH with a neutral charge and low toxicity [11]. It has been shown that proline can protect cells by increasing the water uptake potential and facilitating enzyme activation [12]. The relationship between proline content and abiotic stress tolerance in plants is still not clearly understood. However, there is a consensus among plant researchers that the accumulation of proline is beneficial for plants, especially during recovery from stress. In recent years, various studies have suggested that the exogenous application of proline by foliar spraying may play an important role in enhancing abiotic stress tolerance in plants [13,14,15,16,17]. Furthermore, many reports reveal that different plant species have different responses to drought stress, which are generally dependent on the intensity of the stress and the species of plant [18,19,20]. *Brassica napus* L. is an important oilseed plant globally and the oil extracted from it is used for human and industrial applications due to its fatty acid composition [21,22,23].

Oilseed rape is highly sensitive to drought stress, and global climate change leading to severe and prolonged drought in some parts of the world is expected to reduce the productivity of rapeseed. Drought stress adversely affects germination, seedling establishment, photosynthetic efficiency, mineral uptake, shoot elongation, yield, and quality in rapeseed [23]. It is a problem that needs to be addressed by knowing the targeted pathways and processes. For this reason, the application of exogenous proline in sustainable agriculture practices has emerged as innovative and environment-friendly technology for improving rapeseed productivity. Understanding the role of proline in rapeseed growth and development under drought stress can help the selection of technologies that may be beneficial to regional climate conditions. For example, in Europe, higher temperatures will accelerate the development of winter rapeseed in autumn, making the crop more susceptible to low spring temperatures, but higher temperatures in summer can also cause drought stress and affect plant growth and development [24]. Although the application of exogenous proline effectively reduces the adverse effects of stress, the effect of proline on rapeseed plants depends on the growth stage, the timing and method of application, and the proline concentration. That is why an advanced study on proline metabolism in stress response should be carried out. Thus, the exogenous application of proline may be an effective approach to reducing the adverse effects of water deficit stress; however, the potential role of proline in improving resistance to prolonged drought in rapeseed has not been investigated.

We hypothesize that exogenous proline application will improve drought tolerance and the growth of rapeseed under simulated drought conditions. The objectives of this study were to (1) explore does proline improves shoot growth under water deficit conditions, (2) investigate rape seedling biochemical responses to proline exposure during prolonged drought stress, and (3) determine the effect of proline on plant recovery and survival after watering.

## 2. Results

### 2.1. Options of Different Concentrations of Proline to Improve RWC and Growth Recovery of Drought-Stressed Oilseed Rape

Differences in the RWC of oilseed rape leaves became more pronounced on the 8th day of drought: plants treated with 1 mM proline at 3 and 12.5 mL per pot had RWC of 71% and 73% (mild stress), respectively, whereas drought-only treated plants had RWC of 63% and 52% (high stress). It should be noted that low doses of proline also slightly increased the RWC of leaves of continuously watered plants, i.e., from 83% to 86%. Thus, with a treatment of 1 mM proline at 12.5 mL per pot, plants were less wilted compared to the drought control (Table 1).

The choice of proline concentration and dose to improve the growth of oilseed rape under simulated prolonged drought conditions showed that the most suitable concentration was 1 mM at 12.5 mL per pot (Table 2). It was observed that this dose of proline enhanced the final average weight of recovered seedlings after 4 days of irrigation.

**Figure 1:**
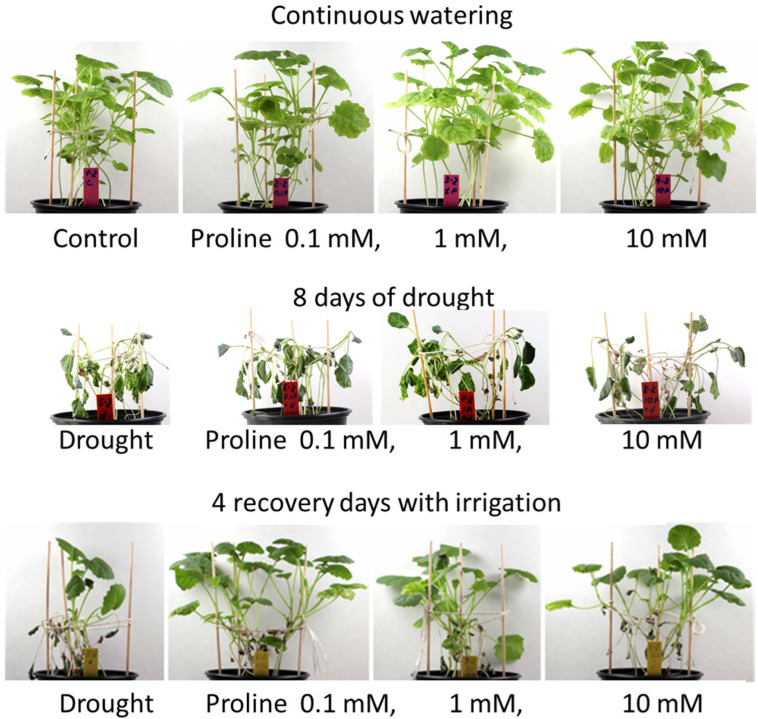
Photograph of *Brassica napus* seedlings exposed to proline (12.5 mL) after 8 days of drought compared to continuously watered plants.

Thus, treatment with 1 mM proline at 12.5 mL per pot was selected for further experiments because it promotes plant RWC and the growth recovery of drought-stressed plants (Figure 1).

### 2.2. Impact of Proline Application on Morphometric Parameters of Oilseed Rape Seedlings Exposed to Drought

Drought stress caused a decrease in fresh and dry weight and the length of rapeseed seedlings. Proline treatment had no reliable effect on seedling length both in continuously watered and drought-stressed plants. Exogenous application of proline improved the weight of drought-stressed oilseed rape: fresh weight was increased by 31% and 9% and dry weight was increased by 20% and 15% on the 4th and 8th day of drought, respectively (Table 3). The best result was that after 8 days of prolonged drought and recovery after 4 days of irrigation, the length and final average fresh weight of the seedlings treated with 1 mM proline were the highest and close to those of the continuously watered control.

### 2.3. Impact of Proline Application on RWC of Prolonged Drought-Stressed Oilseed Rape Leaves

**Figure 2:**
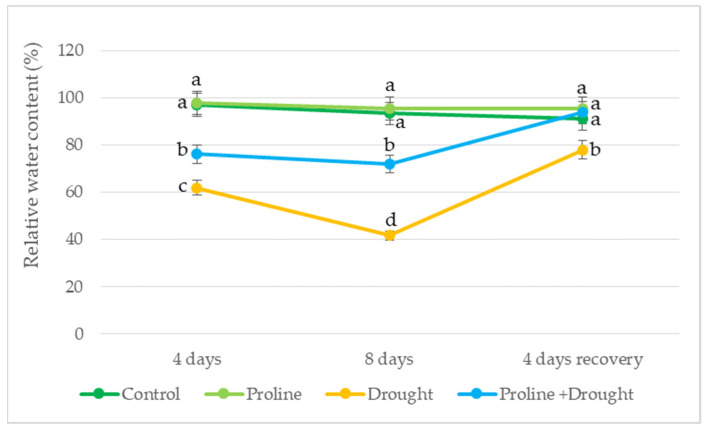
Impact of proline application on RWC of oilseed rape leaves after simulated prolonged drought and recovery by watering. The horizontal axis indicates the duration of prolonged drought and recovery by irrigation. Vertical error bars represent the standard deviation of the mean of three replications (*n* = 3). Different lowercase letters indicate statistically significant differences (*p* < 0.05).

After the first 4 days of drought, the plants were highly stressed according to the Hsiao [25] standard, as shown by the reduction in the RWC of oilseed rape leaves by up to 61% (Figure 2). Proline treatment had a positive effect on the RWC of drought-stressed oilseed rape leaves, increasing it to 79% (moderate stress). Moreover, after 8 days of prolonged drought, the RWC of the proline-treated seedlings was much closer to the irrigated control than that of the drought-stressed plants, which had only 40% RWC. After 4 days of seedling recovery by irrigation, the RWC of the proline-treated plants was close to that of the continuously irrigated control.

### 2.4. Impact of Proline Application on Chlorophyll Content of Drought-Stressed Oilseed Rape Leaves

Analysis of chlorophyll a and b content in oilseed rape leaves showed that after 8 days of drought, the concentration of chlorophyll a decreased to 0.57 mg g^−1^ FW, while that of chlorophyll b decreased to 0.12 mg g^−1^ FW, compared with concentrations in the watered control of 0.99 mg g^−1^ and 0.23 mg g^−1^ FW, respectively. Proline significantly increased chlorophyll accumulation after 4 days of drought vs. untreated drought control, whereas after 8 days of drought, there was no significant difference between chlorophyll accumulations in leaves of these variants. The highest content of total chlorophylls was found in plants exposed to 8 days of prolonged drought followed by 4 days of recovery by irrigation (Table 4).

### 2.5. Impact of Proline Application on Ethylene Emission of Drought-Stressed Oilseed Rape Leaves

**Figure 3:**
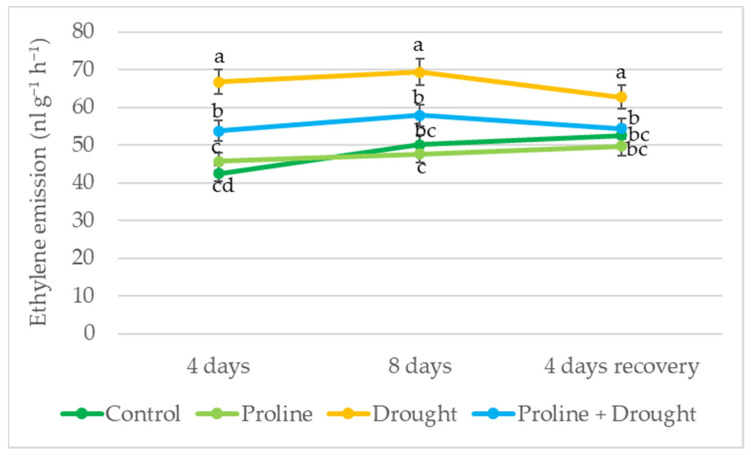
Impact of proline application on ethylene emission of oilseed rape leaves after simulated prolonged drought and recovery by watering. The horizontal axis indicates the duration of prolonged drought and recovery by irrigation. Vertical error bars represent the standard deviation of the mean of three replications (*n* = 3). Different lowercase letters indicate statistically significant differences (*p* < 0.05).

In plants treated with proline, ethylene emission was significantly lower by 30% and 19% compared to only-drought-treated plants on the 4th and 8th day, respectively. After 8 days of drought and 4 days of recovery by watering, ethylene levels in proline-treated plants were reduced to those of the irrigated control (Figure 3).

### 2.6. Impact of Proline Application on H_2_O_2_ Levels of Drought-Stressed Oilseed Rape Leaves

**Figure 4:**
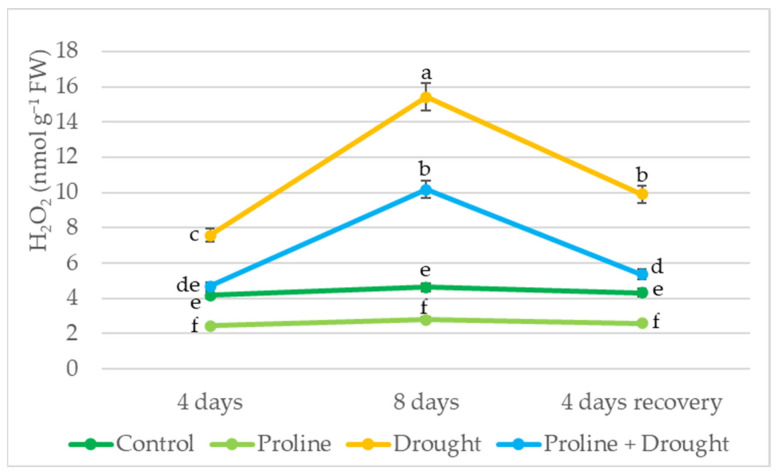
Impact of proline application on H_2_O_2_ level of winter oilseed rape leaves after simulated prolonged drought and recovery by watering. The horizontal axis indicates the duration of prolonged drought and recovery by irrigation. Vertical error bars represent the standard deviation of the mean of three replications (*n* = 3). Different lowercase letters indicate statistically significant differences (*p* < 0.05).

Spraying with exogenous proline significantly reduced H_2_O_2_ content in watered oilseed rape leaf tissues. Impact of exogenous proline manifested on day 4 and day 8 of drought: H_2_O_2_ content decreased to 33% compared to the respective drought control. In addition, after 8 days of drought and 4 days of recovery by watering, H_2_O_2_ levels in proline-treated plants were 2-fold lower (Figure 4).

### 2.7. Impact of Proline Application on MDA Content of Drought-Stressed Oilseed Rape Leaves

**Figure 5:**
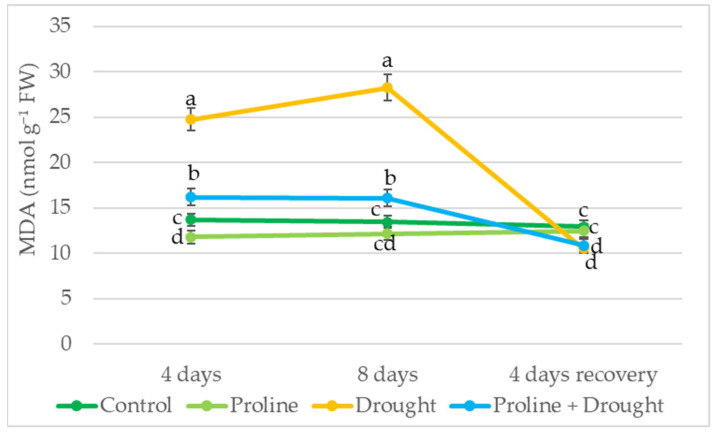
Impact of proline application on MDA content of winter oilseed rape leaves after simulated prolonged drought and recovery by watering. The horizontal axis indicates the duration of prolonged drought and recovery by irrigation. Vertical error bars represent the standard deviation of the mean of three replications (*n* = 3). Different lowercase letters indicate statistically significant differences (*p* < 0.05).

MDA level showed that drought stress significantly increased lipid peroxidation in oilseed rape leaves: after 4 days of drought stress, the amount of MDA in the plants increased 2-fold, and after 8 days even up to 2.5-fold. In plants treated with proline, the MDA content on the 4th and 8th day of the drought was significantly lower by 56% and 75% compared to only-drought-treated plants, respectively. In addition, after 8 days of drought and 4 days of water recovery, both proline + drought-treated plants and only-drought-treated plants had lower MDA content than continuously irrigated plants (Figure 5).

### 2.8. Effects of Exogenous Proline on PM ATPase Activity of Rapeseed Seedlings Exposed to Drought

**Figure 6:**
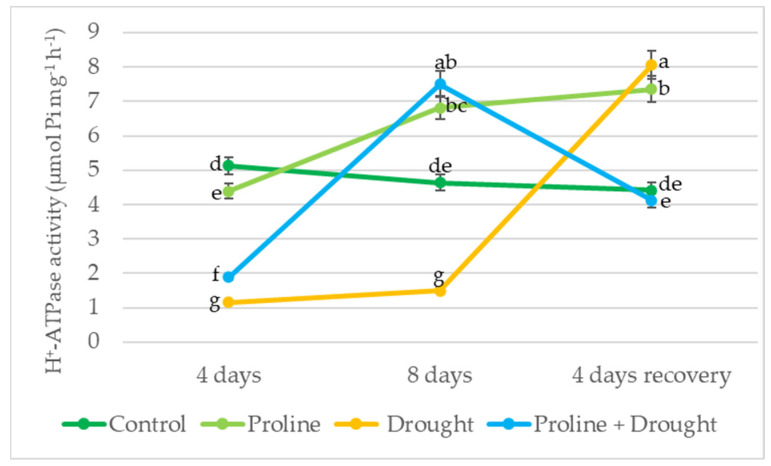
Impact of proline application on PM ATPase activity of winter oilseed rape leaves after simulated prolonged drought and recovery by watering. The horizontal axis indicates the duration of prolonged drought and recovery by irrigation. Vertical error bars represent the standard deviation of the mean of three replications (*n* = 3). Different lowercase letters indicate statistically significant differences (*p* < 0.05).

After 4 days of drought, PM ATPase activity decreased 4-fold in oilseed rape leaf cells. Application with exogenous proline increased the H^+^-ATPase activity of drought-exposed plants by 62% vs. only-drought-treated plants. After 8 days of drought, PM ATPase activity of proline + drought-treated plants increased by 5-fold vs. only-drought-treated plants and approached that of the control after 4 days of recovery by irrigation (Figure 6).

### 2.9. Effect of Exogenous Proline on Endogenous Proline Content of Rapeseed Seedlings Exposed to Drought

**Figure 7:**
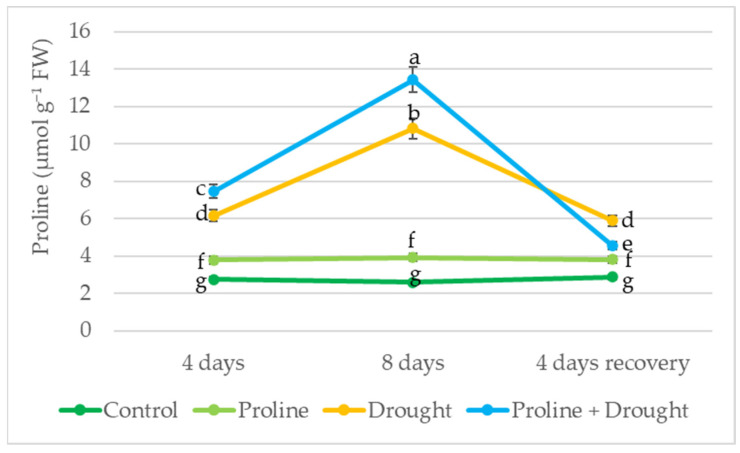
The effect of exogenous proline application and drought stress, on winter rapeseed proline content. Vertical error bars represent the standard deviation of the mean of three replications (*n* = 3). Different lowercase letters indicate statistically significant differences (*p* < 0.05).

The continuously irrigated oilseed rape plants sprayed with proline had an average of 32% more endogenous free proline than the control plants throughout the study period. Drought stress significantly increased proline levels: during the first 4 days of drought, proline levels increased 2-fold, and as the drought progressed, up to 4-fold. Rapeseed plants in the proline + drought variant had 20–23% more free endogenous proline than plants subjected to 8 days of drought alone. In plants treated with proline and exposed to drought for 8 days and 4 days of recovery by irrigation, the endogenous proline content came closer to that of irrigated plants; however, in the only-drought-treated variant, the level of endogenous proline decreased to a lesser extent (Figure 7).

### 2.10. Effect of Exogenous Proline on Survival of Plants

Survival evaluation of winter oilseed rape seedlings after 8 days of simulated drought stress and 12 days of recovery by watering showed that the number of surviving plants was significantly higher (more than 2-fold) after proline treatment compared to only-drought-treated plants (Table 5).

## 3. Discussion

Drought stress of different intensities can trigger different plant responses depending on the plant species [26]. Drought is one of the many factors with a negative effect on the morphology, physiology, and development of plants of the economically important oilseed rape [27,28]. Previous studies have shown that water deficit induces metabolic changes in plants that lead to the accumulation of specific metabolites such as proline [29,30,31]. Moreover, data from the literature indicate that exogenous application of osmoprotectants has shown positive effects on plants growing under drought stress, promoting growth and antioxidant activity [17,32]. In addition, proline plays an essential role in protecting plants from various stresses and helping them recover faster from stress [8]. Previous studies have shown that the relative water content (RWC) of leaves is one of the main indicators of water status and the ability of plants to grow and develop under drought stress [33,34,35]. Thus, RWC indicates the relative water content in leaves and is directly influenced by the soil water content. At the same time, Abdelaal et al. [36] showed that the leaf RWC of barley plants during drought was restored by exogenous proline. We found that exogenous proline affected the relative water content (RWC) in rapeseed leaves under continuously increasing water deficit (simulated drought). Our data showed that drought-treated plants exposed to 1 mM proline had the best leaf water retention (Figure 2). It should be noted that low doses of proline also slightly increased the RWC of leaves of continuously watered plants compared to drought control.

Many authors have provided evidence that drought stress negatively affected the morphological parameters of plants, including the fresh and dry weight of shoots, which decreased under drought stress. Thus, plant biomass formation can be considered an important indicator for drought stress assessment [27,32,35,37]. This coincides with the data of our study, which revealed that drought stress significantly reduced rapeseed plant growth in terms of fresh and dry biomass (Table 3). According to the literature, exogenous proline increased the growth of maize [38] and wheat [39] plants and the dry weight of barley plants [36] under drought conditions. Our research has shown that application of exogenous proline (1 mM) was effective in increasing the fresh and dry mass of shoots, both under normal water supply and under drought stress. The best result was obtained after irrigation recovery of drought-stressed seedlings sprayed with 1 mM proline: the average fresh weight was the highest and close to that of the continuously irrigated control.

There is no doubt that one of the most important changes in metabolic functions during drought is the loss of photosynthetic pigments or a decrease in their synthesis. Numerous studies have reported that drought-induced oxidative stress significantly reduces chlorophyll content in crop plants [28,34,35,40]. This was also confirmed by the data of our study, which showed that moderate and severe drought stress significantly reduced the content of chlorophyll a, b compared to control plants (Table 4). It is important to note that the exposure of plants to exogenous proline before drought significantly improved photosynthetic pigment levels not only throughout the drought but also during plant recovery after the resumption of watering. Other researchers also noted that exposure to exogenous proline improved chlorophyll content in wheat [10], barley [36], and rice [41] plant leaves under drought conditions.

Literature data suggested that ethylene emission in plants increases during drought stress [42]. In our study, ethylene content significantly increased during drought stress. In addition, application with exogenous proline decreased ethylene accumulation vs. only-drought-treated plants, indicating reduced stress levels (Figure 3). After growth recovery by watering, the phytohormone ethylene emission of proline-treated plants remained close to that of the irrigated control. Our data contribute to the suggestion that ethylene may help to remove the inhibitory effect of drought stress on plant growth [43].

Drought may also induce stress responses that result in the accumulation of reactive oxygen species (ROS) such as hydrogen peroxide H_2_O_2_ [8,44]. Our study showed that H_2_O_2_ concentration during 8 days of drought increased 3.3-fold compared to plants grown under irrigation conditions (Figure 4). Similar drought-induced oxidative stress was recorded in oilseed rape plants in studies by other authors [28,34,36]. Excessive concentrations of free radicals, including H_2_O_2_, can cause damage to cell membranes, ion leakage, and osmotic imbalance, so maintaining their level is very important [34,40]. Plant spraying with exogenous proline significantly reduced H_2_O_2_ content in prolonged-drought-stressed rapeseed leaf tissues. In addition, after 4 days of recovery by watering, the H_2_O_2_ level in proline + drought-treated plants decreased by up to 2-fold. These data agreed with the results obtained by Abdelaal [36], where exposure to exogenous proline significantly reduced H_2_O_2_ content in the leaves of barley plants under drought conditions. There are reports in the literature [45,46] characterizing the antioxidant properties of proline in detoxifying ROS. In addition, Rejeb and colleagues [47] in their study concluded that the role of proline as a free radical scavenger is more important than its role as a simple osmolyte.

The stability of cell membranes in drought-stressed plants is adversely affected by ROS, which is evident from the increase in MDA [28,36]. In our study, lipid peroxidation results showed significant membrane damage due to drought. MDA content was elevated in drought-stressed plants and increased with increasing drought time from 1.8- to 2.1-fold that of irrigated plants at 4 and 8 days of drought, respectively (Figure 5). Furthermore, after 8 days of drought and 4 days of recovery by watering, MDA content in proline-treated plants was lower than that in continuously irrigated plants. Similar results showing increased MDA in rapeseed under drought were also obtained by other researchers [28,33,36]. Meanwhile, a significantly lower amount of MDA—1.5- to 1.7-fold—was detected in the cells of plants exposed to exogenous proline before experiencing drought stress, which indicates better stability of the cell membranes of these plants. Similar results where exposure to exogenous proline reduced malondialdehyde levels under drought were obtained in wheat [10], barley [36], and rice [41] plants. This reduction in MDA content could be attributed to the putative role of osmolytes in alleviating the deleterious effects of stress on the structure of cell membranes and activities of PM enzymes, as well as reducing the generation of highly destructive free radicals [38,41,48].

The properties, integrity, and composition of the cell membrane can have a major impact on the activity of the PM ATPase, which displaces protons from the plant cell, creates an electrochemical gradient across the plasma membrane, and plays an important role in various aspects of plant physiology. PM ATPase activity has been reported to be altered by drought in many plant species [49,50]. In our study, PM ATPase activity is significantly reduced by drought stress, a reduction that has been previously reported by other investigators [51,52]. These findings are also in agreement with the results of Mi et al. [53], who showed that Na^+^, K^+^-ATPase, and Ca^2+^, Mg^2+^-ATPase activities of winter rapeseed leave cells gradually decreased with increasing drought severity. In our study, the exogenous application of proline significantly increased PM ATPase activity (from 62 to 400%) compared to drought-exposed, proline-untreated plants (Figure 6). These findings are in line with those of other researchers, who have shown that proline not only protects cells by increasing water uptake potential but also facilitates enzyme activation [12]. In the present study, we showed that the PM ATPase activity of proline-treated oilseed rape recovering from drought stress approached that of control continuously watered plants.

The role of the accumulation of the osmoprotectant proline under drought stress is being actively investigated in order to clarify plant resistance to dehydration. Several reports indicate that under drought stress, plants accumulate higher levels of osmolytes such as proline, which prevent cellular water loss and play an important role in maintaining tissue turgor [54,55]. Our study found that prolonged drought stress significantly increased proline levels, with a 2-fold increase in proline levels during the first 4 days of drought, and as much as a 4-fold increase as the drought progressed (Figure 7). Literature data demonstrate that the exogenous application of proline can increase its endogenous levels in plant tissues exposed to drought stress contributing to the maintenance of drought adaptation in plant tissues [17]. In our research, we confirmed that exogenous proline treatment increased the concentration of endogenous proline in rapeseed leaves under prolonged drought. In addition, it is suggested that the higher levels of proline accumulated in plants exposed to severe and moderate stress conditions may be essential in plant recovery from stress [56]. Our research showed that after watering was renewed, endogenous proline levels of proline + drought-treated plants approached those of irrigated plants.

It was also evident that exogenous proline (1 mM) improved the survival of winter oilseed rape seedlings upon resumption of irrigation after 8 days of drought stress. We estimated that the number of surviving plants after 12 days of recovery was more than 2-fold higher for the proline + drought-treated variant compared to plants exposed only to drought. This is a novel study of the enhanced plant recovery processes after surviving drought. Thus, the response of plants to drought stress is complex and involves many physiological, biochemical, cellular, and molecular changes to ensure plant survival.

## 4. Materials and Methods

### 4.1. Plant Material and Growth Conditions

Oilseed rape seeds (*Brassica napus* L. cv. ‘Visby’) were sown in plastic cubic pots with a peat moss substrate (pH 5.5–6.5). Plants were germinated and grown under controlled conditions of a constant temperature of 23 ± 1 °C, a photoperiod of 16/8 h, a fluorescent light photon flux of 60 µmol m^−2^ s^−1^ at soil level, and 65% humidity in a Climacell plant growing chamber (Medcenter Einrichtungen GmbH). Soil moisture was maintained at ~70%.

### 4.2. Treatments

L-proline aqueous solutions (Roth) were used for seedling spraying at the 3–4 leaf stage (BBCH-scale 13–14 [57]).

**Figure 8:**
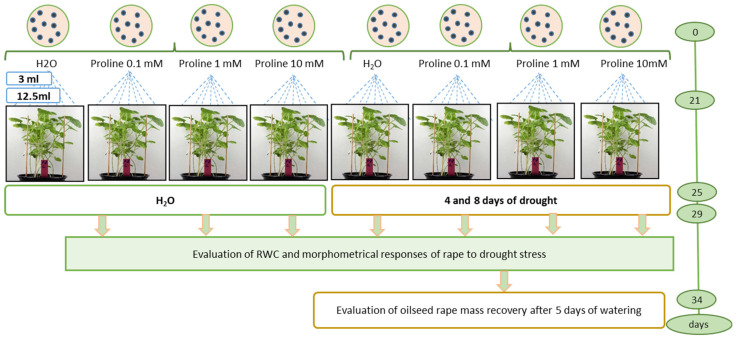
Experimental design of determination of the active proline concentration studies; 3 mL and 12.5 mL–the volume sprayed on oilseed rape plants.

Drought treatment for the drought stress control studies: After 21 days of cultivation, 3–4 leaf-stage seedlings (sprayed with water or 1 mM proline) were subjected to prolonged drought stress for 8 days to reach a high water deficit (Figure 8). During the simulated drought, irrigation was interrupted to allow the soil to dry out gradually. Soil moisture was assessed using a soil moisture meter (Biogrod, China). Irrigation was then resumed and recovery was assessed after 4 days.

### 4.3. Determination of the Active Proline Concentration

To determine the active proline concentration, seeds of oilseed rape were sown in 16 × 3 pots with peat substrate (Figure 8). Each experimental unit consisted of 14 seeds. Pots without proline served as control. Three treatments of the study of proline impact were used: (1) control, H_2_O 3 mL per pot; (2) proline 0.1 mmol 3 mL per pot; (3) proline 1 mmol 3 mL per pot; (4) proline 10 mmol 3 mL per pot; (5) control, H_2_O 12.5 mL per pot; (6) proline 0.1 mmol 12.5 mL per pot; (7) proline 1 mmol 12.5 mL per pot; (8) proline 10 mmol 12.5 mL per pot. The other eight treatments were the same plus drought treatment. Seedlings at the BBCH 13–14 third-fourth leaves stage [57] were foliar sprayed with water solutions of proline and the control was sprayed with water.

### 4.4. Experimental Design of Drought-Stress Control Studies

**Figure 9:**
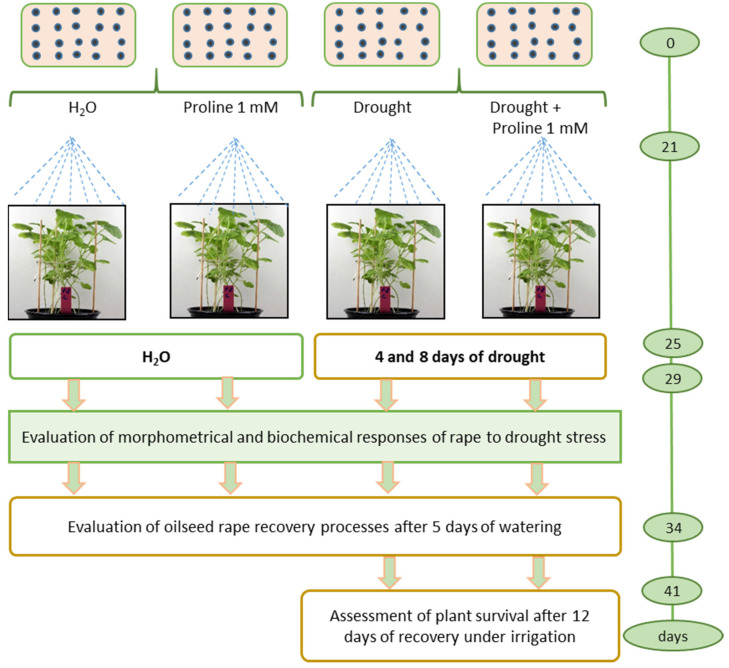
Experimental design of simulated drought-stress control studies.

Plants were sown in plastic cubic pots (15 × 35 cm) (12 pots in total), 60 seeds per pot, in a peat moss substrate. Four pots were used for the experiment, two for rational watering and two for drought simulation, repeated three times with four pots each. Aqueous solutions of L-proline 1 mmol 12.5 mL per pot were sprayed according to the following scheme: (1) control watering, (2) proline and watering, (3) drought, and (4) proline and drought (Figure 9).

### 4.5. Sampling

Plant samples were taken for analysis on three occasions: on the 4th day of the drought (soil moisture 40%), on the 8th day of the drought (soil moisture 20%), and on the 4th day of plant recovery after watering when soil moisture was 70%. The watered plants, used as controls, were sampled at the same time (soil moisture 70%). Shoots of 30 wheat seedlings were sampled for morphometrical measurements. For biochemical analysis, three independent replicates were carried out using the third leaves of rapeseed plants. Freshly harvested samples were used for ethylene emission analysis and pigment measurement. For H^+^-ATPase activity, MDA, H_2_O_2,_ and proline assays, the samples were immediately frozen in liquid nitrogen and stored in a low-temperature freezer (Skadi Green line, EU) at −80 °C until the analysis. Survived seedlings were counted immediately after 41 days of cultivation.

### 4.6. Morphometrical Measurements

Shoot length and fresh and dry mass were taken after 4, 8, and 12 days of growth after treatments using a ruler and balances (Kern EWJ, Bertschikon, Switzerland and Sartorius BP 110S, Songdo, Republic of Korea).

### 4.7. Recovery Evaluation

The same morphometric and biochemical parameters were measured as those after 4 and 8 days of drought.

### 4.8. Survival Evaluation

The plant survival test was carried out by starting to water the plants after 8 days of prolonged drought stress. The test requires a wait of 5–12 days to confirm that winter oilseed rape seedlings are recovering by generating green shoots at the apical meristem growth point in the middle leaf rosette [58]. Thus, plants in each pot were scored as dead or alive after a 12-day recovery period. Plant survival was presented as a percentage of recovered plants.

### 4.9. Relative Water Content (RWC)

To determine RWC, rapeseed leaves were collected and weighed as fresh weight (FW). Then leaves were allowed to fully hydrate on the surface of pure water for 1 day at 4 °C in the dark and their turgid weight (TW) was recorded. The leaves were dried for 2 days at 80 °C in a drying chamber and weighed to determine the dry weight (DW). RWC values were calculated according to the formula: RWC = 100 × (FW − DW)/(TW − DW) [59].

### 4.10. Assessment of Biochemical Parameters

#### 4.10.1. Photosynthetic Pigments

The photosynthetic pigments were extracted from fresh leaves with N, N′-dimethyl-formamide (DMF) (Sigma-Aldrich, St. Louis, MO, USA). Light absorption was measured at 480, 664, and 647 nm. The chlorophyll a/b ratio and chlorophyll a, b content were calculated according to Wellburn [60].

#### 4.10.2. Ethylene

The method of Child et al. [61] was used to evaluate ethylene emission from freshly harvested leaves. Samples with known mass were placed in 40 mL clear glass vials (Agilent technologies, Santa Clara, CA, USA) sealed with PTFE/Si septa caps and incubated for 24 h at 21 °C in darkness. Following incubation, 1 mL of gas sample from each vial was sampled using a gas-tight syringe (Agilent Technologies) and injected into a gas chromatograph equipped with a stainless-steel column (Propac R, Sigma-Aldrich, USA) and hydrogen flame ionization detector. The temperatures of the injector, column, and detector were 110, 90, and 150 °C, respectively. Helium (AGA) was used as the carrier gas. Calibrations were made with an ethylene standard (Messer, Bad Soden, Germany). Results were expressed as nL g^−1^ FW h^−1^.

#### 4.10.3. Hydrogen Peroxide (H_2_O_2_)

Rapeseed leaves (0.5 g) were homogenized using 5% trichloracetic acid (TCA) (Sigma-Aldrich). H_2_O_2_ content in leaves was determined according to [62]. The supernatant was mixed with 10 mM, pH 7.0 potassium phosphate buffer (Alfa Aesar, Haverhill, MA, USA), and 1 M potassium iodide (Alfa Aesar, Haverhill, MA, USA) in a ratio of 1:1:2. The reaction solution was incubated for 30 min at 25 °C in the dark. The absorbance of the supernatant was measured at 390 nm. The amount of H_2_O_2_ was calculated using a standard curve. The results were expressed in µmol g^−1^ FW.

#### 4.10.4. MDA Content

For analysis of lipid peroxidation, MDA leaf material (0.5 g) was homogenized using 5% trichloracetic acid (TCA) (Sigma-Aldrich). The method of Hodges et al. with slight modifications was used to estimate MDA. The homogenates were centrifuged at 13 g for 17 min (centrifuge MPW-351 R) and the supernatant was added to 20% TCA containing 0.5% thiobarbituric acid (TBA) (Alfa Aesar). The homogenate was incubated in a heater at 95 °C for 30 min (Blockthermostat BT 200, Kleinfeld, Labortechnik) and subsequently cooled on ice. The optical density was measured at 532 and 660 nm using a spectrophotometer (Analytik Jena Specord 210 Plus, Analytik Jena, Jena, Germany)). The results were expressed in µmol g^−1^ FW [63].

#### 4.10.5. H^+^-ATPase Activity Assay

The membrane-enriched microsomal fraction was extracted from plant samples. Protein content was measured using the Bradford dye-binding procedure [64] at 595 nm. The H^+^-ATPase activity of the microsomal fraction was evaluated according to the released inorganic phosphate (P_i_) that accumulates as a result of ATP hydrolysis [65]. The color reaction for P_i_ measurement was performed with ammonium molybdate and stannous chloride at 750 nm. The activity of H^+^-ATPase was expressed as μmol P_i_ mg^−1^ of protein h^−1^.

#### 4.10.6. Proline

A color reaction of acidified ninhydrin was used to determine the content of proline [66]. Equal volumes of supernatant of a ground plant material (0.5 g), acetic acid, and acidified ninhydrin were mixed and heated for 1 h 15 min at 108.5 °C in a heater. The formed chromophore was extracted with toluene. The absorbance was read spectrophotometrically at 520 nm using a multi-sample quartz cuvette and Rainbow microplate reader. The corresponding proline content was determined using the standard curve. Calculations were provided using the SLT program (SLT Labinstruments, Viena, Austria). Results were expressed as µmol of proline µmol g^−1^ FW.

### 4.11. Statistical Analysis

Results are presented as mean ± standard deviation (SD) of three independent experiments with at least three replicates. The data were analyzed using analysis of variance (ANOVA). Tukey’s test was performed to test the statistical significance of differences (*p* < 0.05) between means. The Post hoc test Duncan’s Multiple Range Test (DMRT) was used for comparing the means of different groups after performing an analysis of variance (ANOVA).

## 5. Conclusions

In the current study, we determined that exogenous proline improved the tolerance of rapeseed growth to prolonged drought by maintaining water status, by the accumulation of photosynthetic pigments and osmoprotective substances, and by maintaining the stability of the plasma membrane in leaf tissues, and thus, affected the recovery of irrigated rapeseed seedlings after drought stress. This is the first study of the improvement of plant recovery processes after surviving drought. Exogenous proline initiated changes in endogenous proline levels of leave tissues. The accumulation of proline is beneficial for plants, especially during recovery from stress: the number of survived plants after proline treatment was significantly higher (2-fold) than after drought stress alone. The analysis of the stress-mitigating role of exogenous proline leads to the conclusion that it could be proposed for improving plant drought-stress resistance and increasing the yield of oilseed rape products.

## Figures and Tables

**Table 1:** Effect of Proline Treatment on RWC of Leaves with a Continuous Increase in Water Deficit (Simulating Drought).

Treatment (3 mL)	RWC, %	
	4 Days	8 Days
Control, H_2_O	82.3 a	82.5 a
Proline 0.1 mM	81.5 a	81.7 a
Proline 1 mM	82.3 a	80.7 a
Proline 10 mM	84.0 a	85.6 a
Drought	70.1 c	63.2 d
Proline 0.1 mM + Drought	74.7 b	58.8 e
Proline 1 mM + Drought	73.6 b	70.7 c
Proline 10 mM + Drought	70.9 c	59.8 f
Treatment (12.5 mL)		
Control, H_2_O	84.2 a	83.5 a
Proline 0.1 mM	85.2 a	87.2 a
Proline 1 mM	80.7 a	87.3 a
Proline 10 mM	80.1 a	86.8 a
Drought	63.1 d	51.9 e
Proline 0.1 mM + Drought	73.7 b	60.1 d
Proline 1 mM + Drought	74.6 b	72.6 bc
Proline 10 mM + Drought	70.4 c	58.7 d

Mean (±SE) was calculated from three replicates for each treatment. Values in a column with different letters are significantly different at *p* ≤ 0.05 applying Duncan’s Multiple Range Test (DMRT).

**Table 2:** Effect of Proline on the Weight of Oilseed Rape Recovering from Prolonged Drought after 4 Days of Irrigation.

Treatment (3 mL)	Average Weight (g)	
	Fresh	Dry
Control, H_2_O	0.91 ab	0.051 a
Proline 0.1 mM	0.88 b	0.052 a
Proline 1 mM	1.01 a	0.055 a
Proline 10 mM	1.24 a	0.052 a
Drought	0.84 c	0.041 c
Proline 0.1 mM + Drought	0.75 cd	0.045 ab
Proline 1 mM + Drought	0.78 c	0.049 ab
Proline 10 mM + Drought	0.97 ab	0.044 b
Treatment (12.5 mL)		
Control, H_2_O	1.13 a	0.057 a
Proline 0.1 mM	1.26 a	0.056 a
Proline 1 mM	1.47 a	0.059 a
Proline 10 mM	1.35 a	0.057 a
Drought	1.02 b	0.043 c
Proline 0.1 mM + Drought	1.07 ab	0.043 c
Proline 1 mM + Drought	1.18 a	0.055 ab
Proline 10 mM + Drought	1.11 a	0.051 b

Mean (±SE) was calculated from three replicates for each treatment. Values in a column with different letters are significantly different at *p* ≤ 0.05 applying DMRT.

**Table 3:** Effect of Proline (1 mM) on Morphometric Parameters of Oilseed Rape Seedlings (per Plant) under Simulated Drought Conditions.

Treatment	Average Length (cm)			Average Weight (g)					
				Fresh			Dry		
	4 Days	8 Days	4 Days Recovery	4 Days	8 Days	4 Days Recovery	4 Days	8 Days	4 Days Recovery
Control H_2_O	15.26 a	17.27 a	18.00 b	0.68 b	0.74 b	0.77 b	0.045 a	0.045 a	0.046 a
Proline	15.76 a	17.93 a	19.03 a	0.74 a	0.87 a	0.89 a	0.048 a	0.048 a	0.049 a
Drought	14.64 b	14.74 c	15.71 c	0.51 c	0.45 d	0.46 c	0.030 c	0.041 b	0.038 b
Proline + Drought	14.89 ab	15.29 b	17.75 b	0.67 b	0.49 c	0.70 b	0.036 b	0.047 a	0.039 b

Mean (±SE) was calculated from three replicates for each treatment. Values in a column with different letters are significantly different at *p* ≤ 0.05 applying DMRT.

**Table 4:** Effect of Proline Application on Chlorophyll Content of Oilseed Rape Seedlings under Drought Stress.

Treatment	Chlorophyll Contents (mg g^−1^ FW)								
	Chlorophyll a			Chlorophyll b			Chlorophyll a + b		
	4 Days	8 Days	4 Days Recovery	4 Days	8 Days	4 Days Recovery	4 Days	8 Days	4 Days Recovery
Control, H_2_O	0.98 a	0.99 a	0.85 a	0.24 a	0.23 a	0.24 a	1.22 a	1.22 a	1.09 a
Proline	0.97 a	1.02 a	0.85 a	0.25 a	0.24 a	0.25 a	1.22 a	1.23 a	1.11 a
Drought	0.78 c	0.57 c	0.67 b	0.21 a	0.12 b	0.18 b	0.98 c	0.69 c	0.85 b
Proline + Drought	0.93 b	0.62 b	0.89 a	0.21 a	0.13 b	0.22 a	1.13 b	0.75 b	1.10 a

Mean (±SE) was calculated from three replicates for each treatment. Different letters in columns designate statistically significant differences at *p* ≤ 0.05 applying DMRT.

**Table 5:** Effect of proline on *Brassica napus* plant survival after 8 days of prolonged drought followed by 12 days of recovery by irrigation.

Treatment	Number of Survived Plants (%)
Control H_2_O	100.00 a
Proline	100.00 a
Drought	19.79 ± 2.21 b
Proline + Drought	44.10 ± 3.15 c

Mean (±SE) was calculated from three replicates for each treatment. Values in a column with different letters are significantly different at *p* ≤ 0.05 applying DMRT.

## Data Availability

The data supporting the reported results can be found in the archive of scientific reports of the Nature Research Centre.

## References

[1] Della-Marta P.M., Haylock M.R., Luterbacher J., Wanner H. (2007). Doubled length of western European summer heat waves since 1880. J. Geophys. Res. Atmos..

[2] Peña-Ortiz C., Barriopedro D., García-Herrera R. (2015). Multidecadal Variability of the Summer Length in Europe. J. Clim..

[3] Basarin B., Lukić T., Matzarakis A. (2020). Review of Biometeorology of Heatwaves and Warm Extremes in Europe. Atmosphere.

[4] Petrov P., Petrova A., Dimitrov I., Tashev T., Olsovska K., Brestic M., Misheva S. (2018). Relationships between leaf morpho-anatomy, water status and cell membrane stability in leaves of wheat seedlings subjected to severe soil drought. J. Agron. Crop. Sci..

[5] Bandurska H., Niedziela J., Pietrowska-Borek M., Nuc K., Chadzinikolau T., Radzikowska D. (2017). Regulation of proline biosynthesis and resistance to drought stress in two barley (*Hordeum vulgare* L.) genotypes of different origin. Plant Physiol. Biochem..

[6] Verbruggen N., Hermans C. (2008). Proline accumulation in plants: A review. Amino Acids.

[7] Szabados L., Savouré A. (2009). Proline: A multifunctional amino acid. Trends Plant Sci..

[8] Hayat S., Hayat O., Alyemeni M.N., Wani A.S., Pichtel J., Ahmad A. (2012). Role of proline under changing environments. Plant Signal. Behav..

[9] Dar M.I., Naikoo M.I., Rehman F., Naushin F., Khan F.A. (2016). Proline accumulation in plants: Roles in stress tolerance and plant development. Osmolytes and Plants Acclimation to Changing Environment: Emerging Omics Technologies.

[10] Farooq M., Nawaz A., Chaudhry M., Indrasti R., Rehman A. (2017). Improving resistance against terminal drought in bread wheat by exogenous application of proline and gamma-aminobutyric acid. J. Agron. Crop. Sci..

[11] Hossain M.A., Kumar V., Burritt D.J., Fujita M., Mäkelä P. (2019). Osmoprotectant-mediated abiotic stress tolerance in plants. Proline Metabolism and Its Functions in Development and Stress Tolerance.

[12] Caverzan A., Casassola A., Patussi Brammer S., Shanker A.K., Shanker C. (2016). Reactive oxygen species and antioxidant enzymes involved in plant tolerance to stress. Abiotic and Biotic Stress in Plants—Recent Advances and Future Perspectives.

[13] Orsini F., Pennisi G., Mancarella S., Al Nayef M., Sanoubar R., Nicola S., Gianquinto G. (2018). Hydroponic lettuce yields are improved under salt stress by utilizing white plastic film and exogenous applications of proline. Sci. Hortic..

[14] El Moukhtari A., Cabassa-Hourton C., Farissi M., Savouré A. (2020). How Does Proline Treatment Promote Salt Stress Tolerance During Crop Plant Development?. Front. Plant Sci..

[15] Zhang G., Wang Y., Wu K., Zhang Q., Feng Y., Miao Y., Yan Z. (2021). Exogenous Application of Chitosan Alleviate Salinity Stress in Lettuce (*Lactuca sativa* L.). Horticulturae.

[16] Godoy F., Olivos-Hernández K., Stange C., Handford M. (2021). Abiotic Stress in Crop Species: Improving Tolerance by Applying Plant Metabolites. Plants.

[17] Hosseinifard M., Stefaniak S., Ghorbani Javid M., Soltani E., Wojtyla Ł., Garnczarska M. (2022). Contribution of Exogenous Proline to Abiotic Stresses Tolerance in Plants: A Review. Int. J. Mol. Sci..

[18] Kaur G., Asthir B. (2017). Molecular responses to drought stress in plants. Biol. Plant..

[19] Bukhari S.A.H., Peerzada A.M., Javed M.H., Dawood M., Hussain N., Ahmad S. (2019). Growth and Development Dynamics in Agronomic Crops under Environmental Stress. Agronomic Crops.

[20] Daryanto S., Wang L., Jacinthe P.A. (2020). Global synthesis of drought effects on cereal, legume, tuber and root crops production. Agric. Water Manag..

[21] Cartea E., De Haro-Bailón A., Padilla G., Obregón-Cano S., del Rio-Celestino M., Ordás A. (2019). Seed Oil Quality of *Brassica napus* and *Brassica rapa* Germplasm from Northwestern Spain. Foods.

[22] Kordrostami M., Mafakheri M., Hasanuzzaman M. (2020). Rapeseed: Biology and Physiological Responses to Drought Stress. The Plant Family Brassicaceae.

[23] Batool M., El-Badri A.M., Hassan M.U., Haiyun Y., Chunyun W., Zhenkun Y., Jie K., Wang B., Zhou G. (2022). Drought Stress in Brassica napus: Effects, Tolerance Mechanisms, and Management Strategies. J. Plant Growth Regul..

[24] Pullens J.W.M., Sharif B., Trnka M., Balek J., Semenov M.A., Olesen J.E. (2019). Risk factors for European winter oilseed rape production under climate change. Agric. For. Meteorol..

[25] Hsiao T.C. (1973). Plant responses to water stress. Ann. Rev. Plant Physiol..

[26] Seleiman M.F., Al-Suhaibani N., Ali N., Akmal M., Alotaibi M., Refay Y., Dindaroglu T., Abdul-Wajid H.H., Battaglia M.L. (2021). Drought Stress Impacts on Plants and Different Approaches to Alleviate Its Adverse Effects. Plants.

[27] El Sabagh A., Hossain A., Barutcular C., Gormus O., Ahmad Z., Hussain S., Islam M., Alharby H., Bamagoos A., Kumar N. (2019). Effects of drought stress on the quality of major oilseed crops: Implications and possible mitigation strategies—A review. Appl. Ecol. Environ. Res..

[28] Khan M.N., Zhang J., Luo T., Liu J., Ni F., Rizwan M., Fahad S., Hu L. (2019). Morpho-physiological and biochemical responses of tolerant and sensitive rapeseed cultivars to drought stress during early seedling growth stage. Acta Physiol. Plant..

[29] Signorelli S., Dewi J.R., Considine M.J. (2022). Soil Water Content Directly Affects Bud Burst Rate in Single-Node Cuttings of Perennial Plants. Agronomy.

[30] Radzikowska D., Sulewska H., Bandurska H., Ratajczak K., Szymańska G., Kowalczewski P.Ł., Głowicka-Wołoszyn R. (2022). Analysis of Physiological Status in Response to Water Deficit of Spelt (*Triticum aestivum* ssp. *spelta*) Cultivars in Reference to Common Wheat (*Triticum aestivum* ssp. *vulgare*). Agronomy.

[31] Forlani G., Trovato M., Funck D., Signorelli S., Hossain M.A., Kumar V., Burritt D.J., Fujita M., Mäkelä P.S.A. (2019). Regulation of Proline Accumulation and Its Molecular and Physiological Fctions in Stress Defence. Osmoprotectant-Mediated Abiotic Stress Tolerance in Plants: Recent Advances and Future Perspectives.

[32] Semida W.M., Abdelkhalik A., Rady M.O.A., Marey R.A., Abd El-Mageed T.A. (2020). Exogenously applied proline enhances growth and productivity of drought stressed onion by improving photosynthetic efficiency, water use efficiency and up-regulating osmoprotectants. Sci. Hortic..

[33] Akram N.A., Iqbal M., Muhammad A., Ashraf M., Al-Qurainy F., Shafiq S. (2018). Aminolevulinic acid and nitric oxide regulate oxidative defense and secondary metabolisms in canola (*Brassica napus* L.) under drought stress. Protoplasma.

[34] Hasanuzzaman M., Nahar K., Anee T.I., Khan M.I.R., Fujita M. (2018). Silicon-mediated regulation of antioxidant defense and glyoxalase systems confers drought stress tolerance in *Brassica napus* L.. S. Afr. J. Bot..

[35] Sharif P., Seyedsalehi M., Paladino O., Van Damme P., Sillanpää M., Sharifi A. (2018). Effect of drought and salinity stresses on morphological and physiological characteristics of canola. Int. J. Environ. Sci. Technol..

[36] Abdelaal K.A., Attia K.A., Alamery S.F., El-Afry M.M., Ghazy A.I., Tantawy D.S., Hafez Y.M. (2020). Exogenous application of proline and salicylic acid can mitigate the injurious impacts of drought stress on barley plants associated with physiological and histological characters. Sustainability.

[37] Li J.J., Zeng L., Cheng Y., Lu G.Y., Fu G.P., Ma H.Q., Liu Q.Y., Zhang X.K., Zou X.L., Li C.H. (2018). Exogenous melatonin alleviates damage from drought stress in *Brassica napus* L. (rapeseed) seedlings. Acta Physiol. Plant..

[38] Ali Q., Ashraf M., Athar H.U.R. (2007). Exogenously applied proline at different growth stages enhances growth of two maize cultivars grown under water deficit conditions. Pak. J. Bot..

[39] Kamran M., Shahbaz M., Ashraf M., Akram N.A. (2009). Alleviation of drought-induced adverse effects in spring wheat (*Triticum aestivum* L.) using proline as a pre-sowing seed treatment. Pak. J. Bot..

[40] Aslam M.M., Farhat F., Siddiqui M.A., Yasmeen S., Khan M.T., Sial M.A., Khan I.A. (2021). Exploration of physiological and biochemical processes of canola with exogenously applied fertilizers and plant growth regulators under drought stress. PLoS ONE.

[41] Hanif S., Saleem M.F., Sarwar M., Irshad M., Shakoor A., Wahid M.A., Khan H.Z. (2021). Biochemically Triggered Heat and Drought Stress Tolerance in Rice by Proline Application. J. Plant Growth Regul..

[42] Valluru R., Davies W.J., Reynolds M.P., Dodd I.C. (2016). Foliar Abscisic acid-to-ethylene accumulation and response regulate shoot growth sensitivity to mild drought in wheat. Front. Plant Sci..

[43] Dubois M., Van den Broeck L., Inzé D. (2018). The Pivotal Role of Ethylene in Plant Growth. Trends Plant Sci..

[44] Todorova D., Sergiev I., Katerova Z., Shopova E., Dimitrova L., Brankova L. (2021). Assessment of the Biochemical Responses of Wheat Seedlings to Soil Drought after Application of Selective Herbicide. Plants.

[45] Signorelli S., Coitiño E.L., Borsani O., Monza J. (2014). Molecular mechanisms for the reaction between OH radicals and proline: Insights on the role as reactive oxygen species scavenger in plant stress. J. Phys. Chem. B.

[46] Raza A., Charagh S., Abbas S., Hassan M.U., Saeed F., Haider S., Sharif R., Anand A., Corpas F.J., Jin W. (2023). Assessment of proline function in higher plants under extreme temperatures. Plant Biol. J..

[47] Rejeb K.B., Abdelly C., Savouré A. (2014). How Reactive Oxygen Species and Proline Face Stress Together. Plant Physiol. Biochem..

[48] Dawood M.G., Sadak M.S. (2014). Physiological role of glycinebetaine in alleviating the deleterious effects of drought stress on canola plants (*Brassica napus* L.). Middle East J. Agric. Res..

[49] Gong H.J., Chen K.M., Chen G.C., Wang S.M., Zhang C.L. (2003). Drought stress stimulates p-nitrophenyl phosphate hydrolysis rate of the plasma membrane H^+^-ATPase from wheat leaves. Plant Growth Regul..

[50] Feng X., Liu W., Zeng F., Chen Z., Zhang G., Wu F. (2016). K^+^ uptake, H^+^-ATPase pumping activity and Ca^2+^ efflux mechanism are involved in drought tolerance of barley. Environ. Exp. Bot..

[51] Michalak A., Wdowikowska A., Janicka M. (2022). Plant Plasma Membrane Proton Pump: One Protein with Multiple Functions. Cells.

[52] Chang N., Ziwen Z., Yeyun L., Xianchen Z. (2022). Exogenously applied Spd and Spm enhance drought tolerance in tea plants by increasing fatty acid desaturation and plasma membrane H^+^-ATPase activity. Plant Physiol. Biochem..

[53] Mi C., Wang Q., Zhao Y.A., Zhang C.L., Sun C., Liu Z.G., Lin L.B. (2022). Changes in the Differentially Expressed Proteins and Total Fatty Acid Contents in Winter Rapeseed (*Brassica rapa* L.) Leaves under Drought Stress. Russ. J. Plant Physiol..

[54] Blum A. (2017). Osmotic adjustment is a prime drought stress adaptive engine in support of plant production. Plant Cell Environ..

[55] Wang X., Mao Z., Zhang J., Hemat M., Huang M., Cai J., Jiang D. (2019). Osmolyte accumulation plays important roles in the drought priming induced tolerance to post-anthesis drought stress in winter wheat (*Triticum aestivum* L.). Environ. Exp. Bot..

[56] Kauer G., Asthir B. (2015). Proline: A key player in plant abiotic stress tolerance. Biol. Plant..

[57] Meier U., Meier U. (2018). Growth Stages of Mono and Dicotyledonous Plants. BBCH Monograph.

[58] Fiebelkorn D., Rahman M. (2016). Development of a protocol for frost-tolerance evaluation in rapeseed/canola (*Brassica napus* L.). Crop J..

[59] Weng M., Cui L., Liu F., Zhang M., Shan L., Yang S., Deng X.-P. (2015). Effects of Drought stress on antioxidant enzymes in seedlings of different wheat genotypes. Pak. J. Bot..

[60] Wellburn A.R. (1994). The spectral determination of chlorophylls a and b, as well as total carotenoids, using various solvents with spectrophotometers of different resolution. J. Plant Physiol..

[61] Child R.D., Chauvaux N., John K., Van Onckelen H.A., Ulvskov P. (1998). Ethylene biosynthesis in oilseed rape pods in relation to pod shatter. J. Exp. Bot..

[62] Velikova V., Yordanov I., Edreva A. (2000). Oxidative stress and some antioxidant systems in acid rain-treated bean plants: Protective role of exogenous polyamines. Plant Sci..

[63] Hodges D., DeLong J., Forney C., Prange R.K. (1999). Improving the thiobarbituric acid-reactive-substances assay for estimating lipid peroxidation in plant tissues containing anthocyanin and other interfering compounds. Planta.

[64] Bradford M.M. (1976). A rapid method for the quantification of microgram quantities of proteins utilising the principle of protein-dye binding. Anal. Biochem..

[65] Darginavičienė J., Pašakinskienė I., Maksimov G., Rognli O.A., Jurkonienė S., Šveikauskas V., Bareikienė N. (2008). Changes in plasmalemma K^+^ Mg^2+^-ATPase dephosphorylating activity and H^+^ transport in relation to freezing tolerance and seasonal growth of *Festuca pratensis* Huds. J. Plant Physiol..

[66] Bates L.S., Waldren R.P., Teare I.D. (1973). Rapid determination of free proline for water-stress studies. Plant Soil.

